# Repeated remimazolam sedation for interstitial brachytherapy in cervical cancer: a randomized controlled trial

**DOI:** 10.1080/07853890.2026.2740294

**Published:** 2026-10-04

**Authors:** Yanqing Wang, Handan Yang, Qian Gu, Huaiming Wang, Jiaxin Sun, Yiquan Xu, Jinjun Shu, Deling Zeng

**Affiliations:** a Department of Anesthesiology, Sichuan Clinical Research Center for Cancer, Sichuan Cancer Hospital and Institute, Sichuan Cancer Center, University of Electronic Science and Technology of China, Chengdu, China; b Department of Anesthesiology, Xichang People’s Hospital, Xichang, China; c Health Management Center, Sichuan Clinical Research Center for Cancer, Sichuan Cancer Hospital and Institute, Sichuan Cancer Center, University of Electronic Science and Technology of China, Chengdu, China

**Keywords:** Remimazolam, propofol, repeated sedation, adverse events, sleep quality

## Abstract

**Background:**

Evidence regarding repeated remimazolam administration remains limited. We hypothesized that remimazolam would be associated with fewer adverse events than propofol while maintaining stable sedative requirements across three repeated sedation sessions.

**Methods:**

Patients with cervical cancer undergoing three fractions of interstitial brachytherapy were randomized to propofol or remimazolam. The primary outcome was the proportion of sedation episodes with at least one prespecified intraoperative adverse event across three sessions. Secondary outcomes included sedative dose, hemodynamics, and sleep quality assessed using the Pittsburgh Sleep Quality Index (PSQI) and Athens Insomnia Scale (AIS).

**Results:**

A total of 100 participants were randomized, of whom 91 completed all three prespecified sedation sessions and were included in the primary per-protocol analysis (46 in the propofol group and 45 in the remimazolam group). Across 138 propofol and 135 remimazolam episodes, overall adverse events were less frequent with remimazolam (17.78% vs. 59.42%; RR, 0.30; 95% CI, 0.18–0.49; *p* < 0.001). Respiratory depression (3.70% vs. 26.09%; RR, 0.14; 95% CI, 0.04-0.53; *p* = 0.004) and hypotension (8.15% vs. 41.30%; RR, 0.20; 95% CI, 0.09-0.42; *p* < 0.001) were also lower with remimazolam. Remimazolam requirements remained stable across sessions (third vs. first: mean difference, 0.19 mg; 95% CI, −0.78 to 1.15; *p* = 0.846). No significant between-group differences were observed in PSQI or AIS scores.

**Conclusion:**

Across three consecutive sedation sessions for interstitial brachytherapy, remimazolam was associated with fewer respiratory and hemodynamic adverse events than propofol, while sedative requirements remained stable across the three sessions.

**Trial registration:**

Chinese Clinical Trial Registry (identifier: ChiCTR2500108294).

## Introduction

Repeated sedation is required in various clinical settings involving serial diagnostic or therapeutic procedures. Interstitial brachytherapy is an essential component of treatment for locally advanced cervical cancer and is commonly delivered in multiple fractions. Because applicator placement and interstitial needle insertion are invasive and painful, deep sedation or anesthesia is frequently required for each treatment session [1,2]. The need to repeat these procedures within a relatively short treatment period makes consistent sedative efficacy and cardiorespiratory stability particularly important.

Propofol is widely used for procedural sedation because of its rapid onset and reliable sedative effect [3]. However, it is associated with dose-dependent respiratory depression, hypotension, and injection pain [4,5]. In addition, propofol has been reported to induce rewarding effects and subjective euphoria, raising concerns about its potential for abuse and dependence [6,7]. More importantly, repeated administration of propofol may be associated with the development of tolerance, Previous evidence has shown that 26% of children receiving repeated propofol sedation for proton radiation therapy developed tolerance [8]. These considerations highlight the need for alternative agents that can maintain stable sedation across repeated procedures without increasing respiratory or hemodynamic risk.

Remimazolam is a novel ultrashort-acting benzodiazepine characterized by rapid onset, rapid recovery, organ-independent metabolism, and reversibility with flumazenil [9–12]. Compared with propofol, remimazolam has been associated with greater hemodynamic stability and fewer respiratory complications in several procedural and general-anesthesia settings [13–16]. However, most previous randomized studies have evaluated a single exposure, and randomized evidence regarding repeated remimazolam sedation across serial procedures remains limited. It is therefore uncertain whether its sedative requirements and adverse-event profile remain stable over consecutive treatment sessions.

Regarding the effects of anesthetic sedation on sleep, short-term propofol administration appears to have only a limited impact on sleep architecture [17,18]. However, repeated exposure to propofol may be associated with unfavorable sleep-related effects. Available evidence suggests that repeated or prolonged propofol use can disrupt the natural sleep–wake cycle and may compromise overall sleep quality [19]. In contrast, short-term remimazolam administration appears to exert relatively little influence on postoperative sleep rhythm, and some studies suggest that it may offer advantages over propofol in this respect [20–22].

This randomized controlled trial compared remimazolam with propofol across three consecutive deep-sedation sessions in patients undergoing interstitial brachytherapy for cervical cancer. We hypothesized that remimazolam would be associated with fewer respiratory and hemodynamic adverse events than propofol while maintaining stable sedative requirements across the three procedures.

## Methods

### Ethics and registration

The study protocol was approved by the Ethics Committee for Medical Research and New Medical Technology of Sichuan Cancer Hospital (SCCHEC-02-2025-114). This trial was prospectively registered in the Chinese Clinical Trial Registry on August 27, 2025 (registration number: ChiCTR2500108294), before the first participant was enrolled in October 2025. The study was conducted in accordance with the Declaration of Helsinki and reported according to the Consolidated Standards of Reporting Trials (CONSORT) 2025 statement. Written informed consent was obtained from all patients before the initiation of any protocol-specified procedures.

### Participants

Patients with cervical cancer who required three fractions of interstitial brachytherapy at Sichuan Cancer Hospital from October 2025 to January 2026 were enrolled.

### Inclusion criteria

(1) Patients with cervical cancer scheduled to undergo three fractions of interstitial brachytherapy; (2) American Society of Anesthesiologists (ASA) grade of I or II prior to anesthesia; (3) Body mass index (BMI) ranging from 18 to 30 kg/m^2^.

### Exclusion criteria

(1) Declined to participate in the study; (2) Had a history of severe cardiac, cerebral, pulmonary, hepatic, renal, or metabolic diseases; (3) Had a documented history of adverse anesthesia recovery; (4) Electrocardiogram (ECG) showing heart rate < 50 beats/min; (5) Had an active upper respiratory tract infection within the preceding 2 weeks; (6) Preoperative hypertension (systolic BP > 180 mmHg and/or diastolic BP > 110 mmHg) or hypotension (<90/60 mmHg); (7) Had neuromuscular disorders or psychiatric conditions; (8) Were diagnosed with anemia (hemoglobin below the normal range); (9) Had a history of alcohol abuse or dependence on opioids/sedative-hypnotics; (10) Anticipated or previous difficult airway; (11) Had known allergy to lipid emulsions, benzodiazepines, or opioids; (12) Interval between consecutive procedures <48 h; (13) Pittsburgh Sleep Quality Index (PSQI) score >7 on the day before the procedure.

### Rule‑out criteria

(1) Completed only once or twice interstitial brachytherapy; (2) Perioperative serious adverse events or procedural complications.

### Randomization, allocation concealment, and blinding

The randomization sequence was generated in a 1:1 ratio using the random allocation module of SPSS version 27.0 by an independent researcher who was not involved in participant recruitment, treatment administration, outcome assessment, or statistical analysis. The treatment assignments were placed in sequentially numbered, opaque, sealed envelopes by the same independent researcher.

Potentially eligible patients were screened by a study investigator who had no access to the randomization sequence. After written informed consent had been obtained and eligibility had been confirmed, the next sequentially numbered envelope was opened to determine treatment allocation. The administering anesthesiologist was then informed of the allocation and implemented the assigned sedation protocol.

Participants, surgeons, outcome assessors, and data analysts were blinded to treatment allocation throughout the trial. The anesthesiologist responsible for preparing and administering the study drugs could not be blinded because propofol and remimazolam differed in appearance and preparation. The administering anesthesiologist was not involved in postoperative outcome assessment or statistical analysis.

### Sample size calculation

The sample size was calculated based on the incidence of intraoperative adverse effects. According to previous studies about hysteroscopy demonstrating intraoperative adverse effects incidence at 7.5% with remimazolam and 36.6% with propofol [23,24]. Using PASS version 2021 with a signiffcance level (α) of 0.05 (two-sided) and power (β) of 0.8, 38 patients per group were required. Accounting for a dropout rate of 20%, 48 patients per group were planned. Ultimately, 50 patients with cervical cancer were included in each group.

### Anesthesia protocol

All patients adhered to an 8-hour fasting period for solids and a 4-hour fasting period for clear fluids prior to each interstitial brachytherapy, and no premedication was administered. A forearm vein was accessed for intravenous cannula insertion. Sodium lactate Ringer’s solution was infused intravenously. At the same time, the heart rate (HR), electrocardiogram (ECG), and peripheral oxygen saturation (SpO_2_) were constantly monitored. Also, every 3 min, noninvasive blood pressure (NIBP) was monitored automatically.

During the procedure, all patients received supplemental oxygen (5 L/min) *via* face mask. Sufentanil (Yichang Renfu Pharmaceutical Co., Ltd.) 0.1 µg/kg and ondansetron 4 mg were administered over two minutes before sedative administration. Thereafter, in group P, patients received 1.5 mg/kg of propofol (Yangtze River Pharmaceutical Co., Ltd.) intravenously for more than 60 s during each interstitial brachytherapy session, followed by continuous infusion at 2–6 mg/kg/h for sedation maintenance. In group R, patients received 0.3 mg/kg of remimazolam (Jiangsu Hengrui Pharmaceutical Co., Ltd.) intravenously for more than 60 s during each interstitial brachytherapy session, followed by continuous infusion at 0.2–1.0 mg/kg/h to maintain sedation. Throughout the procedure, sedation depth was continuously monitored using the Modified Observer’s Assessment of Alertness/Sedation (MOAA/S) scale from induction onward. The procedure commenced upon achieving adequate sedation (MOAA/S score ≤ 2). Subsequent assessments were performed at 5-minute intervals until full recovery. If MOAA/S was above 2, a 0.5 mg/kg bolus of propofol was administered accordingly in group P, or a 0.1 mg/kg bolus of remimazolam was administered as additional sedation in group R.

Intraprocedural hemodynamic and respiratory management followed a standardized protocol. For hypotension accompanied by HR < 55 bpm, intravenous ephedrine (5–10 mg IV) was administered. Isolated hypotension with normal HR was treated with metaraminol (0.2–0.5 mg IV). Hypertensive episodes managed with urapidil (10–15 mg IV), while tachycardia (HR > 110 bpm) was treated with esmolol (10 mg IV). Significant bradycardia (HR < 50 bpm) without hypotension warranted atropine (0.3–0.5 mg IV). Respiratory depression prompted immediate intervention with 10 L/min oxygen and jaw-thrust maneuver. Persistent hypoxemia necessitated the suspension of the procedure for mask ventilation, with tracheal intubation reserved for refractory cases.

### Outcome measures

The primary outcome was the proportion of sedation episodes in which at least one prespecified adverse event occurred across the three sedation sessions. The prespecified intraoperative adverse events included respiratory depression, hypotension, hypertension, bradycardia, tachycardia, and body movement.

Each sedation session was treated as one observation. A sedation episode was counted once for the overall adverse-event outcome if one or more prespecified adverse events occurred during that session. When multiple types of adverse events occurred during the same episode, they were recorded separately in the corresponding adverse-event categories but were counted only once in the overall adverse-event outcome. Events occurring during different sedation sessions in the same participant were treated as repeated observations.

Respiratory depression was defined as SpO_2_ < 94% persisting for more than 10 s. Hypotension was defined as MAP <65 mmHg or a decrease of more than 20% from baseline persisting for more than 10s [25]. Hypertension was defined as MAP >105 mmHg or an increase of more than 20% from baseline persisting for more than 10s [26]. Bradycardia and tachycardia were defined as HR <50 beats/min and HR >110 beats/min, respectively.

### Measures

 

1. Mean arterial pressure (MAP) and HR were recorded at the following time points: 2 minutes prior to anesthesia induction (T0), vaginal speculum insertion (T1), first needle insertion (T2), end of the operation (T3), and awakening (T4).
2. Postoperative adverse effects, such as the incidence of dizziness, postoperative nausea and vomiting (PONV).
3. Sleep Quality assessments, PSQI scores and Athens Insomnia Scale (AIS) was recorded at three time points (preoperative day 1, postoperative day 1, and postoperative day 7).

### Data analysis

Statistical analyses were performed using SPSS version 27.0 (IBM Corp., Armonk, NY), with all continuous variables initially evaluated for normality using the Kolmogorov-Smirnov test. Normally distributed data were expressed as means ± standard deviation (SD) and analyzed using independent Student’s *t*-tests for between-group comparisons or paired *t*-tests for within-group pre-post analyses. Non-normally distributed data were presented as medians (interquartile range) analyzed using Mann-Whitney U tests for between-group comparisons or Wilcoxon signed-rank tests for within-group analyses. Categorical variables were expressed as frequencies (percentages) and compared using chi-square or Fisher’s exact tests as appropriate.

Repeated outcomes were analyzed using generalized estimating equations (GEE) to account for correlations among observations from the same participant. Participant ID was specified as the clustering variable, an exchangeable working correlation structure was used, and robust sandwich standard errors were applied. Repeated binary outcomes were analyzed using GEE models with a binomial distribution and log link function, with treatment effects for the principal binary outcomes expressed as risk ratios (RRs) with 95% confidence intervals (CIs). Continuous repeated outcomes were analyzed using GEE models with a Gaussian distribution and identity link function. Depending on the outcome, treatment group, sedation session or measurement time, and the corresponding group-by-session or group-by-time interaction were included as model effects.

The primary analysis was performed in the per-protocol population, defined as participants who completed all three prespecified sedation sessions. Participants who underwent only one or two fractions of interstitial brachytherapy were not included in the primary per-protocol analysis.

As a sensitivity analysis, all randomized participants were analyzed according to their original treatment allocation using all available session-level observations. GEE models were used to account for within-participant correlation and differences in the number of observed sedation sessions. No outcome values were imputed for sessions in which interstitial brachytherapy and sedation were not performed because no corresponding sedation episode existed.

All statistical tests were two-sided, and a *p* value < 0.05 was considered statistically significant.

## Results

A total of 168 patients with cervical cancer were screened. 68 were excluded: Patients with BMI > 30 kg/m^2^ (*n* = 4); Patients with communication barriers(*n* = 2); Patients with ECG showing a heart rate < 50 bpm (*n* = 8); Hypertensive patients with uncontrolled blood pressure (*n* = 6); Patients with systolic blood pressure < 90 mmHg during screening (*n* = 2); Patients with predicted or previous difficult airways (*n* = 2); Patients scheduled to undergo two fractions of interstitial brachytherapy (*n* = 36); PSQI score >7 on the day before the procedure (*n* = 8). A total of 100 patients were randomized equally to group P (*n* = 50) and group R (*n* = 50). 4 patients in group P and 5 patients in group R were lost during follow-up due to completed less than three fractions of interstitial brachytherapy. A total of 46 patients in group P and 45 in group R were finally included in the analysis (Figure 1). The demographic characteristics of the patients were presented in Table 1, and no statistically significant differences were observed between the groups. (*p* > 0.05, Table 1).

**Figure 1.**
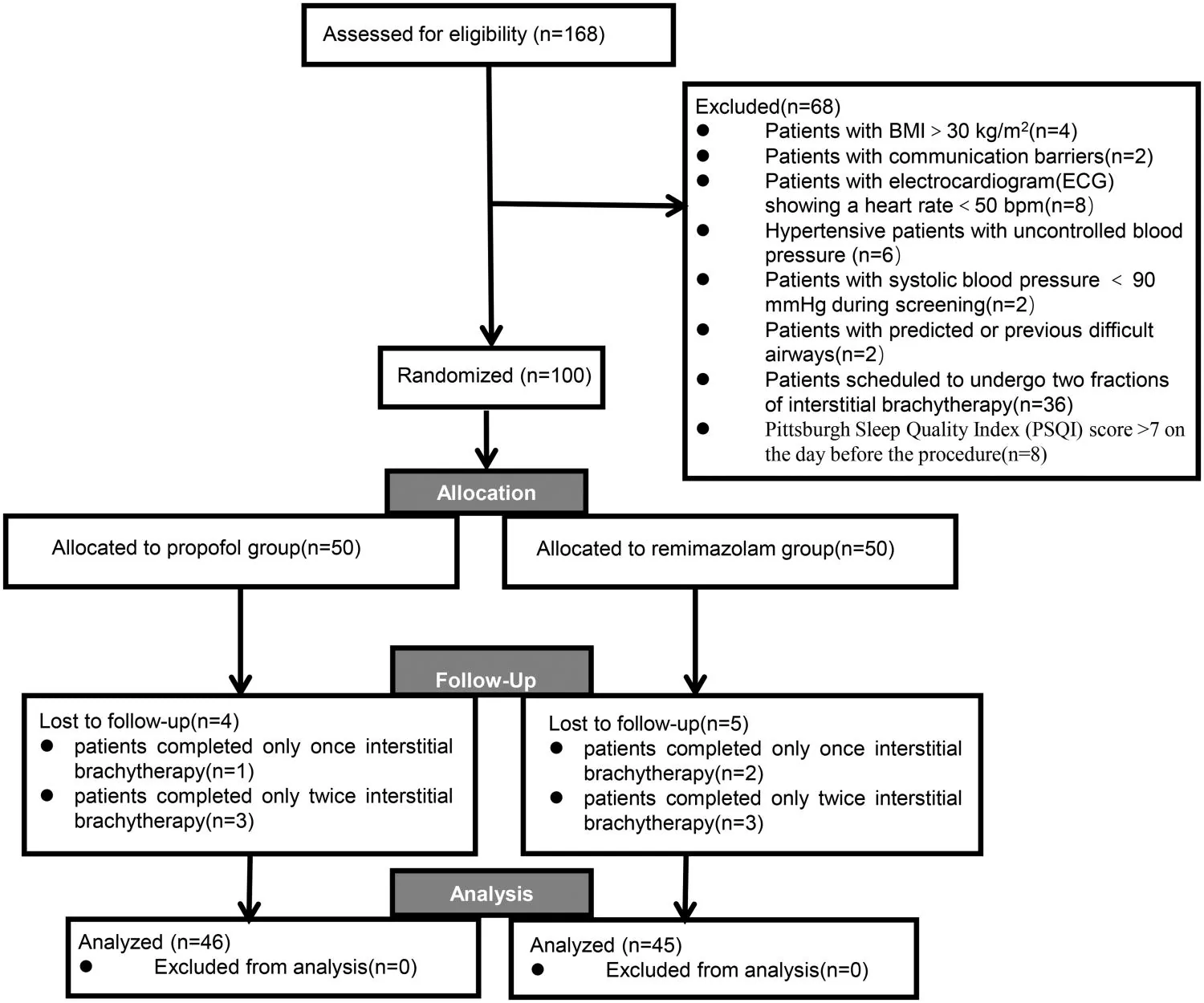
Patient flowchart with CONSORT guidelines. Group P, propofol group; Group R, remimazolam group. BMI, body mass index; ECG, electrocardiogram; PSQI, Pittsburgh Sleep Quality Index; CONSORT, Consolidated Standards of Reporting Trials.

**Table 1.** Patient characteristics.

Variable	Group P (*n* = 46)	Group R (*n* = 45)	*p* Value
Age (years)	57.43 ± 8.95	57.04 ± 10.74	0.851
Weight (kg)	56.20 ± 6.78	54.42 ± 8.07	0.259
Height (cm)	154.61 ± 4.66	154.84 ± 7.12	0.852
BMI (kg/m^2^)	23.54 ± 2.87	22.68 ± 2.82	0.152
Baseline Characteristics			
MAP (mmHg)	83.98 ± 7.61	82.40 ± 8.96	0.367
HR (beats/min)	82.02 ± 9.95	84.11 ± 8.55	0.286
SpO_2_ (%)	98.00 (97.00, 98.00)	98.00 (96.00, 98.00)	0.753
ASA Grade (*n*, %)			
Grade I	39, 84.78%	39, 86.67%	0.822
Grade II	7, 15.22%	6, 13.33%	
Comorbidities (*n*, %)			
Hypertension	5, 10.87%	4, 8.89%	0.767
Respiratory Diseases	0, 0%	0, 0%	–
Cardiovascular Disease	0, %	0, %	–

Variables presented as mean ± standard deviation, median (interquartile range), or number, percentage (%). Group P, propofol group; Group R, remimazolam group. BMI, Body Mass Index; MAP, Mean Arterial Pressure; HR, heart rate; SpO_2_, peripheral oxygen saturation; ASA, American Society of Anesthesiologists.

### The incidence of adverse events

Across the three sedation sessions, the overall incidence of adverse events was significantly lower in group R than in group P (17.78% vs. 59.42%; RR, 0.30; 95% CI, 0.18–0.49; *p* < 0.001). Compared with group P, group R showed significantly lower incidences of respiratory depression (3.70% vs. 26.09%; RR, 0.14; 95% CI, 0.04–0.53; *p* = 0.004) and hypotension (8.15% vs. 41.30%; RR, 0.20; 95% CI, 0.09–0.42; *p* < 0.001). Bradycardia occurred in 5/138 sedation episodes (3.62%) in the propofol group and 0/135 episodes (0%) in the remimazolam group. The absolute risk reduction associated with remimazolam was 22.38% for respiratory depression and 33.16% for hypotension. No significant between-group differences were observed in the incidences of hypertension (2.22% vs. 1.45%, *p* = 0.635), body movements (3.70% vs. 1.45%, *p* = 0.240), or postoperative nausea and vomiting (2.96% vs. 2.17%, *p* = 0.681). No tachycardia occurred in either group. These results are presented in detail in Table 2.

**Table 2.** Episode-level incidence of adverse events across three sedation sessions.

Variable	Group P (*n* = 46)	Group R (*n* = 45)	RR (95% CI)	*p* Value
Adverse events (*n*/*N*, %)	82/138, 59.42%	24/135, 17.78%	0.30 (0.18–0.49)	<0.001
Respiratory depression	36/138, 26.09%	5/135, 3.70%	0.14 (0.04–0.53)	0.004
Hypotension	57/138, 41.30%	11/135, 8.15%	0.20 (0.09–0.42)	<0.001
Hypertension	2/138, 1.45%	3/135, 2.22%	1.53 (0.21–11.01)	0.635
Bradycardia	5/138, 3.62%	0/135, 0.00%	–	–
Tachycardia	0/138, 0.00%	0/135, 0.00%	–	–
Body movement	2/138, 1.45%	5/135, 3.70%	2.56 (0.52–12.50)	0.240
PONV (*n*/*N*, %)	3/138, 2.17%	4/135, 2.96%	1.36 (0.28–6.73)	0.681

Each participant underwent three sedation sessions. A total of 138 sedation episodes in Group P (propofol group) and 135 sedation episodes in Group R (remimazolam group) were included in the analysis. Data are presented as the number of sedation episodes with the specified event/total number of sedation episodes (%). PONV, Postoperative nausea and vomiting; RR, risk ratios; CI, confidence intervals.

A sensitivity analysis was additionally performed using all available sedation-session observations from all 100 randomized participants according to their original treatment allocation. This analysis included 145 observed sedation episodes in the propofol group and 143 in the remimazolam group. The results were consistent with those of the primary per-protocol analysis. Remimazolam remained associated with a lower episode-level risk of overall adverse events (RR, 0.27; 95% CI, 0.16–0.45; *p* < 0.001), respiratory depression (RR, 0.13; 95% CI, 0.04–0.50; *p* = 0.003), and hypotension (RR, 0.19; 95% CI, 0.09–0.41; *p* < 0.001) (Supplementary Table S1).

### Outcomes across three repeated sedation sessions

In group P, no significant differences were observed among the first, the second, and third sessions in respiratory depression, hypotension, hypertension, bradycardia, operative duration, or total propofol dosage (all *p* > 0.05; Table 3). No tachycardia occurred during any of the three sessions.

**Table 3.** Comparison of adverse event across the three sedation sessions in the two groups.

Variable	First session	Second session	Third session	*p* Value
Group P (*n* = 46)				
Respiratory Depression (*n*, %)	12, 26.09%	10, 21.74%	14, 30.43%	0.674
Hypotension (*n*, %)	21, 45.65%	19, 43.48%	17, 41.30%	0.699
Hypertension (*n*, %)	0, 0.00%	1, 2.17%	1, 2.17%	0.602
Bradycardia (*n*, %)	2, 4.35%	1, 2.17%	2, 4.35%	0.813
Tachycardia (*n*, %)	0, 0.00%	0, 0.00%	0, 0.00%	–
Group R (*n* = 45)				
Respiratory Depression (*n*, %)	1, 2.22%	3, 6.67%	1, 2.22%	0.618
Hypotension (*n*, %)	4, 8.89%	3, 6.67%	4, 8.89%	0.906
Hypertension (*n*, %)	0, 0.00%	1, 0.00%	2, 4.44%	0.360
Bradycardia (*n*, %)	0, 0.00%	0, 0.00%	0, 0.00%	–
Tachycardia (*n*, %)	0, 0.00%	0, 0.00%	0, 0.00%	–

Variables presented as number, percentage (%). Group P, propofol group; Group R, remimazolam group.

In group R, the incidences of respiratory depression, hypotension, and hypertension remained comparable across the three sessions, and no bradycardia or tachycardia was observed (all *p* > 0.05; Table 3).

Sedative requirements remained stable across the three procedures. In the remimazolam group, the estimated difference in total dose between the third and first sessions was 0.19 mg (95% CI, −0.78 to 1.15 mg), with no significant overall session effect (*p* = 0.846). Similarly, propofol requirements showed no evidence of progressive dose escalation (third vs first: mean difference, −0.49 mg; 95% CI, −4.54 to 3.56 mg; overall *p* = 0.828).

The sensitivity analysis of sedative requirements yielded findings consistent with the primary analysis. In the remimazolam group, there was no evidence of progressive dose escalation across the three sessions based on the available observations; the estimated difference in total dose between the third and first sessions was 0.20 mg (95% CI, −0.75 to 1.15 mg), with no significant overall session effect (*p* = 0.774) (Supplementary Table S2 and Table 4).

**Table 4.** Comparison of operative duration and sedative dosage across the three sedation sessions in the two groups.

Variable	First session	Second session	Third session	Second vs first session, MD (95% CI)	Third vs first session, MD (95% CI)	*p* Value
Group P (*n* = 46)						
Operative duration (s)	306.93 ± 12.91	311.37 ± 13.06	307.59 ± 12.08	4.43 (−4.65, 13.52)	0.65 (−7.36, 8.66)	0.611
Total dosage of propofol (mg)	101.52 ± 2.67	102.45 ± 2.58	101.03 ± 2.46	0.92 (−3.38, 5.23)	−0.49 (−4.54, 3.56)	0.828
Group R (*n* = 45)						
Operative duration (s)	309.80 ± 13.07	310.53 ± 13.75	310.02 ± 12.73	0.51 (−9.22, 10.24)	−0.22 (−9.53, 9.09)	0.987
Total dosage of remimazolam (mg)	20.26 ± 0.48	20.29 ± 0.54	20.07 ± 0.50	0.22 (−0.55, 1.00)	0.19 (−0.78, 1.15)	0.846

Variables presented as mean ± standard deviation. Group P, propofol group; Group R, remimazolam group. MD, mean difference; CI, confidence interval. Mean differences represent the estimated change in the second or third sedation session relative to the first session and are presented with 95% confidence intervals. The *p* values represent the overall effect of sedation session.

### Comparison of MAP and HR at each time point during repeated sedation

MAP and HR at T0 were comparable between group P and group R during all three sedation sessions. MAP differed significantly between the two groups mainly at T1 (*p* = 0.023 in the first session, *p* < 0.001 in the second and third sessions) and T2 (*p* < 0.001 in the first session, *p* = 0.002 in the second session, and *p* = 0.006 in the third session) across the three sessions, whereas no significant between-group differences were observed at T3 or T4.

Significant differences in HR were observed at several subsequent time points, most consistently at T2, T3 and T4 (all *p* < 0.001 in the first, the second, and the third sessions). HR at T1 differed significantly between the two groups only during the second and third sedation sessions (*p* = 0.503 in the first session, *p* = 0.038 in the second session, and *p* < 0.001 in the third session) (Figure 2).

**Figure 2.**
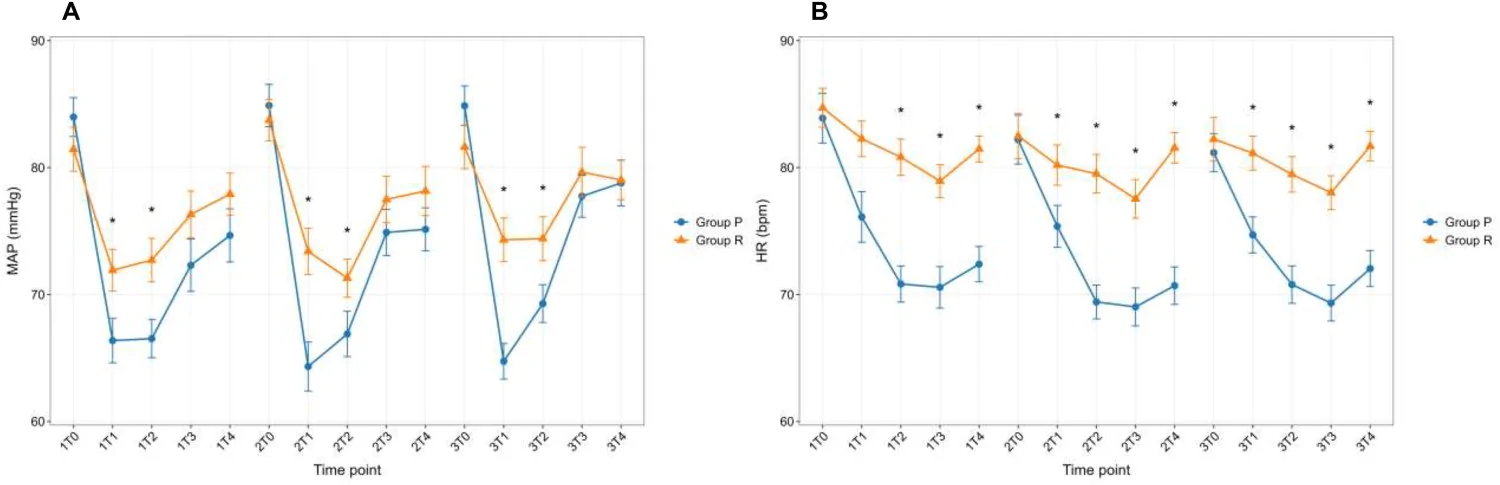
**Changes in mean arterial pressure and heart rate during the three repeated sedation sessions.** (A) Changes in mean arterial pressure; (B) changes in heart rate. The prefixes 1, 2, and 3 indicate the first, second, and third sedation sessions, respectively. T0, 2 min before sedation induction; T1, vaginal speculum insertion; T2, insertion of the first interstitial needle; T3, completion of needle implantation; T4, awakening. Group P, propofol group; Group R, remimazolam group; MAP, mean arterial pressure; HR, heart rate. *p < 0.05 for the comparison between group P and group R.

No progressive deterioration in MAP or HR was observed at each time point across the three repeated sedation sessions in either group P or group R.

### Sleep quality outcomes

There were no significant differences in PSQI or AIS scores between group P and group R at preoperative day 1, postoperative day 1, or postoperative day 7 (all *p* > 0.05) (Table 5). No significant group-by-time interaction was observed for either PSQI (*p* = 0.438) or AIS scores (*p* = 0.135).

**Table 5.** Comparison of PSQI and AIS scores between the two groups.

Variable	Group P (*n* = 46)	Group R (*n* = 45)	Rank-biserial r (95% CI)	*p* Value
PSQI scores				
Preoperative day 1	6.50 (5.00, 7.00)	6.00 (5.50, 7.00)	0.01 (−0.22, 0.23)	0.953
Postoperative day 1	6.00 (5.00, 7.00)	6.00 (5.50, 7.00)	−0.01 (−0.24, 0.22)	0.941
Postoperative day 7	6.00 (5.00, 7.00)	6.00 (5.50, 7.00)	0.04 (−0.19, 0.26)	0.726
AIS scores				
Preoperative day 1	3.50 (1.75, 5.00)	3.00 (2.00, 5.00)	0.05 (−0.19, 0.28)	0.655
Postoperative day 1	3.00 (1.75, 4.00)	3.00 (2.50, 4.00)	0.09 (−0.14, 0.32)	0.433
Postoperative day 7	3.50 (2.00, 5.00)	3.00(2.00, 5.00)	0.02 (−0.22, 0.25)	0.894

Variables presented as median (interquartile range). PSQI, Pittsburgh Sleep Quality Index; AIS, Athens Insomnia Scale; CI, confidence interval; Group P, propofol group; Group R, remimazolam group.

## Discussion

In this randomized clinical trial of patients undergoing three consecutive sessions of interstitial brachytherapy for cervical cancer, remimazolam was associated with fewer respiratory and hemodynamic adverse events than propofol across the repeated procedures, particularly respiratory depression and hypotension. More importantly, remimazolam requirements remained stable across all three sedation sessions, with no evidence of progressive dose escalation, while operative duration also remained comparable across sessions. No significant between-group differences or differential changes over time were detected in questionnaire-assessed sleep outcomes during follow-up. These findings suggest that remimazolam can provide consistent sedative requirements across repeated short-interval procedures, with the lower cardiorespiratory event rates observed relative to propofol being maintained over three consecutive sedation sessions.

Remimazolam is rapidly metabolized by nonspecific tissue esterases to an inactive metabolite, with its elimination being relatively independent of hepatic and renal function [11]. This pharmacokinetic profile may be advantageous in patients with impaired organ function. Previous studies have also suggested that remimazolam is safe and effective in high-risk patients with ASA physical status III or IV [27]. These characteristics may be clinically relevant in patients with cervical cancer undergoing repeated brachytherapy, particularly because treatment for locally advanced disease frequently includes systemic anticancer therapy that may affect hepatic or renal function. This may partly explain why the incidence of adverse events was significantly lower in the remimazolam group than in the propofol group in the present study (17.78% vs. 59.42%, *p* < 0.001).

The remimazolam induction dose of 0.3 mg/kg used in the present study was selected on the basis of accumulated clinical evidence. This dose was supported by the recent meta-analysis evaluating remimazolam for procedural sedation [28], as well as by findings from our previous clinical study [13]. The lower incidences of respiratory depression and hypotension observed with remimazolam were consistent with previous evidence, including recent systematic reviews and meta-analyses demonstrating comparable sedative efficacy to propofol during procedural sedation together with lower risks of hypotension and respiratory complications [29,30]. Evidence specific to gynaecologic procedural sedation has shown a similar pattern, with comparable sedation success but lower incidences of hypotension and respiratory depression with remimazolam than with propofol [31]. Remimazolam was associated with an absolute risk reduction of 22.38% for respiratory depression and 33.16% for hypotension. In patients undergoing serial interstitial brachytherapy, each additional procedure represents another opportunity for respiratory or hemodynamic adverse events to occur. Maintaining lower event rates across repeated sedation sessions may therefore reduce the need for interventions such as airway maneuvers or vasoactive treatment.

Nevertheless, most previous studies have evaluated remimazolam during single procedural exposures, whereas the present study examined its use across three consecutive sedation sessions. This repeated-procedural setting provides an important context for interpreting the stability of sedative requirements observed in our study.

A particularly important finding in this repeated-procedural setting was the stability of sedative requirements across consecutive sessions. Sedation duration and drug requirements remained stable across all three sessions in both groups, with no evidence of progressive dose escalation. In particular, the estimated difference in remimazolam dose between the third and first sessions was only 0.19 mg (95% CI, −0.78 to 1.15 mg). Previous studies have reported that repeated deep sedation with propofol for proton radiation therapy in young children, delivered multiple times (mean, 19 sessions), led to tolerance in 26% of patients, although the increase in propofol dose was minimal [8]. In the present study, no apparent propofol tolerance was observed after three repeated propofol administrations, probably because of the relatively short sedation duration and limited number of exposures. Importantly, repeated remimazolam administration was associated with neither progressive dose escalation nor an apparent increase in the frequency of respiratory or hemodynamic adverse events across the three sessions. These findings are clinically relevant for patients undergoing fractionated interstitial brachytherapy, in whom multiple sedation procedures are required within a relatively short treatment period, and suggest that remimazolam can provide consistent sedative requirements over three consecutive procedures.

Research has consistently shown that a higher prevalence of sleep disorders in women [32]. In women undergoing hysteroscopic surgery, remimazolam was associated with less mental fatigue on the first postoperative day compared with propofol, although overall postoperative insomnia scores were similar between groups [20]. In the present study, no significant differences in PSQI or AIS scores were observed between the remimazolam and propofol groups at any assessed time point, and three consecutive sessions of remimazolam sedation were not associated with significant changes in PSQI or AIS scores at 1 or 7 days after completion of the procedures. Although benzodiazepines are known to alter sleep architecture by prolonging non-rapid eye movement sleep and suppressing slow-wave and rapid eye movement sleep [33]. Nevertheless, evidence regarding the effects of remimazolam on perioperative sleep remains limited. Recent studies have suggested that remimazolam may have less influence than propofol on circadian rhythm-related biomarkers, potentially contributing to a lower risk of postoperative sleep disturbance [21]. A recent systematic review and meta-analysis in older patients also reported a lower incidence of sleep disturbances with remimazolam than with propofol [34]. However, findings have not been entirely consistent. In elderly patients undergoing total joint arthroplasty, although modest improvements were observed in sleep recovery and overall sleep quality scores [35]. More broadly, a recent systematic review and meta-analysis of pharmacological interventions for postoperative sleep in older adults included remimazolam among the evaluated agents but indicated that the evidence supporting most interventions remains limited [36]. Taken together, current evidence regarding the effects of remimazolam on perioperative sleep remains inconclusive. Future studies involving longer surgical procedures and more objective sleep assessments are warranted to better characterize the effects of remimazolam on postoperative sleep quality.

This study had several limitations. First, the relatively small sample size may have limited the precision of the effect estimates. Second, this single-center study enrolled a selected, relatively lower-risk population, including only patients with ASA physical status I-II and BMI 18–30 kg/m^2^, while excluding those with anemia or clinically relevant baseline sleep disturbance (PSQI > 7). These criteria may limit the generalizability of both the cardiorespiratory and sleep-related findings. Third, the administering anesthesiologist could not be blinded because of differences in drug appearance and preparation. Although sedation management followed predefined criteria and standardized protocols, performance bias cannot be completely excluded. Fourth, previous sedative exposure may have influenced subsequent sessions despite the required minimum interval of 48 h between procedures. Fifth, sleep quality was assessed only using the PSQI and AIS, without objective measures such as actigraphy or polysomnography; therefore, subtle changes in sleep patterns or architecture may have been missed. Finally, only three sedation sessions were evaluated over a relatively short period, and the absence of progressive dose escalation should not be interpreted as definitive evidence that pharmacological tolerance would not develop with more prolonged or frequent remimazolam exposure.

## Conclusion

Across three consecutive sedation sessions for interstitial brachytherapy, remimazolam was associated with fewer respiratory and hemodynamic adverse events than propofol. Remimazolam requirements remained stable across all three sessions, demonstrating dose stability during repeated administration.

## Supplementary Material

CONSORT_2025editable checklist.docx

Supplementary_Table_S2_Sensitivity_analysis_of_operative_duration_and_sedative_requirements_using_all_available_observations.docx

Supplementary_Table_S1_Sensitivity_analysis_of_adverse_events_using_all_available_sedation_session_observations.docx

## Data Availability

The data that support the findings of this study are available on request from the corresponding author, J Shu. The data are not publicly available due to their containing information that could compromise the privacy of research participants.
