# Supplementary material for: Repeated remimazolam sedation for interstitial brachytherapy in cervical cancer: a randomized controlled trial
Source: Ann Med. 2026 Oct 4;58(1):2740294. doi: 10.1080/07853890.2026.2740294 (PMC13644436; doi:10.1080/07853890.2026.2740294)
Supplement: Supplementary file 1 [file IANN_A_2740294_SM8670.docx]

**Table S1** Sensitivity analysis of adverse events using all available sedation-session observations

| Variable | Group P | Group R | RR (95% CI) | *p* value |
| --- | --- | --- | --- | --- |
| Adverse events (n/N, %) | 85/145 (58.62%) | 23/143 (16.08%) | 0.27 (0.16–0.45) | <0.001 |
| Respiratory depression | 37/145 (25.52%) | 5/143 (3.50%) | 0.13 (0.04–0.50) | 0.003 |
| Hypotension | 58/145 (40.00%) | 11/143 (7.69%) | 0.19 (0.09–0.41) | <0.001 |
| Hypertension | 3/145 (2.07%) | 3/143 (2.10%) | 0.97 (0.16–5.90) | 0.971 |
| Bradycardia | 5/145 (3.45%) | 0/143 (0.00%) | - | 0.026 |
| Tachycardia | 0/145 (0.00%) | 0/143 (0.00%) | - | - |
| Body movement | 2/145 (1.38%) | 5/143 (3.50%) | 2.54 (0.52–12.43) | 0.251 |
| PONV (n/N, %) | 3/145 (2.07%) | 4/143 (2.80%) | 1.35 (0.27–6.68) | 0.713 |

This available-case sensitivity analysis included all 100 randomized participants according to their original treatment allocation and all observed sedation sessions (145 episodes in Group P and 143 episodes in Group R). Overall adverse events were defined as the occurrence of at least one prespecified intraoperative adverse event during a sedation episode; PONV was not included in this composite. RRs compare Group R with Group P and were estimated using generalized estimating equations with a binomial distribution and log link function, participant ID as the clustering variable, an exchangeable working correlation structure, and robust sandwich standard errors. Group P, propofol group; Group R, remimazolam group. PONV, postoperative nausea and vomiting; RR, risk ratio; CI, confidence interval.
