# Supplementary material for: Repeated remimazolam sedation for interstitial brachytherapy in cervical cancer: a randomized controlled trial
Source: Ann Med. 2026 Oct 4;58(1):2740294. doi: 10.1080/07853890.2026.2740294 (PMC13644436; doi:10.1080/07853890.2026.2740294)
Supplement: Supplementary file 2 [file IANN_A_2740294_SM8671.docx]

**Supplementary Table S2** Sensitivity analysis of operative duration and sedative requirements using all available observations

| Variable | First session | Second session | Third session | Second vs first session, MD (95% CI) | Third vs first session, MD (95% CI) | *p* value |
| --- | --- | --- | --- | --- | --- | --- |
| Group P |  |  |  |  |  |  |
| Operative duration (s) | 307.08 ± 85.85 (n = 50) | 311.76 ± 87.44 (n = 49) | 307.59 ± 82.84 (n = 46) | 4.15 (-4.43, 12.72) | 0.51 (-7.39, 8.40) | 0.616 |
| Total dosage of propofol (mg) | 102.70 ± 18.36 (n = 50) | 103.62 ± 17.88 (n = 49) | 101.03 ± 16.89 (n = 46) | 1.02 (-3.03, 5.07) | -0.71 (-4.69, 3.28) | 0.750 |
| Group R |  |  |  |  |  |  |
| Operative duration (s) | 307.32 ± 83.07 (n = 50) | 310.60 ± 90.41 (n = 48) | 309.80 ± 88.69 (n = 45) | 1.09 (-8.10, 10.28) | 0.11 (-9.05, 9.27) | 0.966 |
| Total dosage of remimazolam (mg) | 20.08 ± 3.36  (n = 50) | 20.40 ± 3.60  (n = 48) | 20.26 ± 3.25  (n = 45) | 0.26 (-0.47, 0.99) | 0.20 (-0.75, 1.15) | 0.774 |

Data are presented as mean ± standard deviation, with the number of observed sessions shown in parentheses. Mean differences (MDs) and 95% CIs were estimated using generalized estimating equations with a Gaussian distribution and identity link function, participant ID as the clustering variable, an exchangeable working correlation structure, and robust sandwich standard errors. The first session was the reference. The *p* value represents the overall effect of sedation session. No outcome values were imputed for sessions in which brachytherapy and sedation were not performed. MD, mean difference; CI, confidence interval.
